# The Country's Children

**Published:** 1908-05-23

**Authors:** 


### The Hospital

A Journal of

The Medical Sciences and Hospital Administration.

New Series. No. 62, Vol. III. [No. 1134, Vol. XLIV.] SATURDAY, MAY 23, 1908.

### The Country's Children.

The public notice given to the future of the country's children is of most hopeful augury. Judging by the space devoted to this subject in the daily press, we may draw the gratifying conclusion that in interest it does not compare unfavourably with problems touching the future of the country's beer and tariff principles—a circumstance of no mean significance. In the course of one week the President of the Local Government Board received a deputation urging the claims of a pure-milk supply and delivered an address to the members of the National Conference on Infantile Mortality, of which he is the president, while in the House of Commons the Lord Advocate moved the second reading of the Children's Bill.

Since the explosion of Koch's thesis that bovine tuberculosis is not directly communicable to man, we have known that the purity of the milk supply has to be considered with reference to two prime contaminations: first, the tubercle bacillus—an intrinsic contamination for the most part, being derived from mammary tuberculosis in the cow; and, secondly, the organisms capable of producing gastro-enteritis in infants—an extrinsic contamination, reaching the milk in its passage from the dairy to the child's stomach. There is little doubt that of these two contaminations the latter is by far the more lethal in its effects, for though primary tuberculosis of the alimentary canal is common enough to call urgently for prevention, still, by comparison with the massacre of infants resulting from gastro-enteritis in a hot summer, the deaths resulting from the ingestion of tuberculous milk are as nothing. This circumstance is unfortunate enough, for the elimination of the tuberculous cow from the dairy herd is by no means an impracticable ideal, though it may take time to compass this result; but the prevention of extrinsic contamination cannot be more than partially achieved by any legislative enactment. It implies an individual caution which no amount of legal procedure can instil in the housewife's mind, a caution which can only be inculcated by a slow process of education. It is an unlucky thing that the fluid which is a vital necessity to the suckling should be at the same time one which is an almost perfect medium for the cultivation of microbic parasites; but so it is, and there is good ground for fearing that the yearly toll laid upon infant life by summer diarrhœa will continue to be exacted with more or less rigour until this knowledge is made the common property of mothers in our cities. The current belief, salutary enough in itself, that sour milk is dangerous for infants, is not the whole truth; indeed it is only true at all in so far as sourness indicates age and contamination; for the bacillus of lactic acid fermentation has been shown to be harmless—indeed, cultures of this organism are actually being employed for therapeutic purposes, because it exerts an inhibitory effect upon other common organisms of intestinal putrefaction. There is a good deal of evidence that summer diarrhœa depends upon a specific organism or group of organisms, and that the propagation of the infection is carried out by flies. The house-fly, whether alive or dead, is a common inhabitant of the milk-basin even in the houses of the well-to-do, but in the houses of the poor a half-emptied tin of sweetened condensed milk, left carelessly open as it often is, forms nothing less than an efficient fly-trap.

These considerations show that the protection of infant life, in so far as the milk-supply is concerned, can never be effected by control of the dairies, be this never so efficient. Such control is undoubtedly called for, but more remains. This vital remainder is education. Nothing is more pitiable to the physician's eye than the spectacle of a young mother of the poorer class, whose caligraphy is past criticism, but whose ignorance in the care of infants is as abysmal as the ordinary bachelor's. Mr. Burns, with that bluff common sense which we have learned to expect from him, put the business very plainly and well to his audience at Caxton Hall. In his judgment, a mother ought not to sublet her maternity, for she cannot, if she would, sub-contract the source of the infant's life, which she alone commands, to any bottle or to any artificial food. After giving birth to her child, the mother must be at home as long as possible; she must be encouraged to nurse her child, avoiding all artificial foods and employing the natural food if it be forthcoming; if not, then she must use the best substitute—namely, milk—milk suitable to the child, milk from cows kept clean and suitably fed, and free from disease. The milk should be from clean dairies, milked by clean hands, put into clean vessels, stored in clean places, and conveyed to the baby's mouth in vessels that should not be the microbe-traps too often used. Mr. Burns preaches a robust and lively gospel, and the truth is in him. Many difficulties lie in the way of his ideal, but the least of them is maternal ignorance, for all the apparatus needed for its dissipation is at hand. Girls' schools, especially among the class which cannot afford trained nurses, should be schools for the teaching of true motherhood, and the schedule of studies ought to include a grounding in the laws governing bacterial diseases.

